# Automated multi-slice extracellular and patch-clamp experiments using the WinLTP data acquisition system with automated perfusion control

**DOI:** 10.1016/j.jneumeth.2012.04.008

**Published:** 2012-06-15

**Authors:** William W. Anderson, Stephen M. Fitzjohn, Graham L. Collingridge

**Affiliations:** a MRC Centre for Synaptic Plasticity, School of Physiology and Pharmacology, University of Bristol, University Walk, Bristol BS8 1TD, UK; b WinLTP Ltd., Bristol BS6 6NS, UK; c Department of Brain & Cognitive Sciences, College of Natural Sciences, Seoul National University, Gwanak-gu, Seoul 151-746, South Korea

**Keywords:** Automated perfusion, Multi-slice, Brain slices, Protocol scripting, Drug discovery, Long-term potentiation (LTP)

## Abstract

WinLTP is a data acquisition program for studying long-term potentiation (LTP) and other aspects of synaptic function. Earlier versions of WinLTP (*J. Neurosci. Methods*, 162:346–356, 2007) provided automated electrical stimulation and data acquisition capable of running nearly an entire synaptic plasticity experiment, with the primary exception that perfusion solutions had to be changed manually. This automated stimulation and acquisition was done by using ‘Sweep’, ‘Loop’ and ‘Delay’ events to build scripts using the ‘Protocol Builder’. However, this did not allow automatic changing of many solutions while running multiple slice experiments, or solution changing when this had to be performed rapidly and with accurate timing during patch-clamp experiments. We report here the addition of automated perfusion control to WinLTP. First, perfusion change *between* sweeps is enabled by adding the ‘Perfuse’ event to Protocol Builder scripting and is used in slice experiments. Second, fast perfusion changes *during* as well as *between* sweeps is enabled by using the Perfuse event in the protocol scripts to control changes between sweeps, and also by changing digital or analog output during a sweep and is used for single cell single-line perfusion patch-clamp experiments. The addition of stepper control of tube placement allows dual- or triple-line perfusion patch-clamp experiments for up to 48 solutions. The ability to automate perfusion changes and fully integrate them with the already automated stimulation and data acquisition goes a long way toward complete automation of multi-slice extracellularly recorded and single cell patch-clamp experiments.

## Highlights

► Almost complete automation of synaptic plasticity and pharmacology experiments is achieved by adding automated perfusion to stimulation/acquisition protocol scripting. ► For multi-slice experiments, automated perfusion control is helpful when there is a need to change many solutions on many separately perfused slices. ► For single cell patch-clamp experiments, automated perfusion control is helpful when many solutions have to be rapidly changed with accurate timing. ► For slice and single cell, single-line automated perfusion, up to 16 different solutions can be delivered. ► For single cell, triple-line automated perfusion with stepper control, up to 48 different solutions can be delivered.

## 1 Introduction

One of the goals of electrophysiological data acquisition program developers is to increase the ease of running single experiments, and to increase the number of experiments that can be run simultaneously. This includes when complex stimulation and perfusion changes have to be performed very quickly such as during a patch-clamp experiment, and when relatively simple stimulation and perfusion changes have to be performed at similar times on multiple slice experiments where making the correct manipulation can become very demanding.

WinLTP is a Windows data acquisition program designed to investigate long-term potentiation (LTP), long-term depression (LTD) and other synaptic responses and has already been described in Anderson and Collingridge (2007). The capabilities required for basic LTP and LTD experiments include alternating two-input extracellular pathway stimulation, LTP induction by single train, theta burst, and primed burst stimulation, and LTD induction by low frequency stimulation. Central to WinLTP is the ‘Protocol Builder’, which enables scripts to be written containing ‘Loop’ (with counter), ‘Delay’, and stimulation/acquisition ‘Sweep’ events that, in turn, enable complex stimulation and data acquisition experiments to be performed. Protocol flow can be changed while the experiment is running. Furthermore, WinLTP is multi-tasking in that gap-free Axon Binary Files can be generated by the ‘Continuous Acquisition’ task, which can run simultaneously with the sweep generating Protocol Builder task. WinLTP also provides on-line measurement of slope, peak amplitude, population-spike amplitude, average amplitude, area, rise time, decay time and duration of synaptic waveforms, and cell input resistance and patch electrode series resistance.

In Anderson and Collingridge (2007), the Protocol Builder scripts were continuous loops regularly producing single pulse sweeps. Induction of synaptic plasticity by electrical stimulation was performed either by manually evoking single train sweeps for LTP or repetitive single pulse sweeps for LTD, or by using a ‘Run’ event section that could produce more complex induction stimulation such as repetitive train sweeps separated by single pulse sweeps to induce in vitro kindling.

In this paper we discuss sequential scripts where once a stable baseline has been determined, one click of the mouse causes the rest of the stimulation for the whole experiment to run. Furthermore, we have added a ‘Perfuse’ event to the Protocol Builder that allows changes of perfusion solution between sweeps. The Perfuse event produces digital or analog output which controls perfusion controllers which turn perfusion valves on and off. This change in perfusion *between* sweeps can also be coupled by changes in perfusion solution *during* sweeps.

Most electrophysiological data acquisition programs can produce perfusion changes during sweeps by changing digital or analog output during the sweeps, but changes between sweeps is usually done manually. There are, to our knowledge, three other electrophysiological programs that can also produce perfusion changes between sweeps, Heka's PatchMaster (www.heka.com), Molecular Device's pClamp (www.moleculardevices.com), and Lohmann Research's SynchroBrain (www.lohres.com).

WinLTP and its predecessor, the LTP Program (Anderson and Collingridge, 2001), have been used in over 350 research papers. A free ‘Basic Mode’ version of WinLTP and a commercial ‘Advanced Mode’ version of WinLTP, with the Protocol Builder and automated perfusion control, are available at www.winltp.com.

## 2 Methods

WinLTP (version 2.00) is a Windows program that runs on Windows XP, Vista and 7. It was written with Embarcadero (Borland) C++ Builder using Win32 VCL components. WinLTP uses National Instruments M- and X-Series boards and Molecular Devices Digidata 1322A and 1320A boards. However, the automated perfusion control in WinLTP described here is currently only available on the National Instruments boards. Other electrophysiological data acquisition programs that use National Instruments boards include AxographX (www.axographx.com), GePulse, mPhys, Nclamp, NeuroRighter, QUB, Serf Software Electrophy, Slice, WinWCP, WinEDR, and custom in-house software using Igor (www.wavemetrics.com), LabView (www.ni.com), and MatLab (www.mathworks.com).

WinLTP is designed to control the 8 channel VC^3^-8 perfusion controller from ALA Scientific (www.alascience.com), the 8 channel ValveLink8.2 controller from Automate Scientific (www.autom8.com), the 8 channel VC-8P controller from Warner Instruments (www.warnerinstruments.com), and the 16 channel PC-16 controller from Bioscience Tools (www.biosciencetools.com).

All these controllers can be controlled by digital output (1 bit/channel or valve, and more than one valve can be open at once—which is important for pre-flush perfusion), or by analog output (usually 1 V/channel or valve for 8 channel controllers and 0.5 V/channel or valve for 16 channel controllers, and only one valve can be open at once). Most of the controllers can also be controlled by 4-bit binary input to control 8, 15 or 16 channels with only one valve open at a time.

In WinLTP, the two extracellular stimulating outputs (‘S0’ and ‘S1’) go to digital outputs 0 and 1 of the high-speed Port0 on M- and X-Series boards and are used to trigger stimulus isolators. There are an additional 4 sync outputs produced during a sweep that also go to Port0, and these are used for Fast0 and Fast1 4-bit digital control of 8, 15 or 16 channels on the perfusion controllers.

The M- and X-Series boards also contain two static or ‘slow-speed’ 8-bit digital output ports, Ports 1 and 2, and these are used for Slow0 and Slow1 8-bit digital control of one or two perfusion controllers. The 8-bit binary output from one port can control 4 pre-flush channels (using 2 valves/channel), or 8 standard channels (1 valve/channel). The 16-bit binary output from both ports together can control 8 pre-flush channels or 16 standard channels. These slow-speed ports are controlled in WinLTP by directly writing to them from within the program rather than having them being written to by outputting a stream of data as is the case for the high-speed Port0. Therefore, while timing for high-speed Port0 output is accurate to the microsecond and is therefore suitable for accurately changing outputs during as well as between sweeps, the timing for slow-speed Ports 1 and 2 are usually accurate to milliseconds or tens of milliseconds and are therefore only accurate enough for perfusion changes between sweeps. Also, by using 1-bit per valve rather than binary digital control, a single physical controller can, in essence, be two logical controllers. For example, Slow0 Port1 digital output can control 4 valves of an 8 valve controller, and Slow1 Port2 digital output can control the other 4 valves of the 8 valve controller.

The intracellular analog stimulation output ‘IC0’ goes to AnalogOut0 of the M- and X-Series boards and usually controls the command voltage of a patch-clamp amplifier. The analog ‘IC1’ output goes to AnalogOut1 and can control 8 or 16 channels and valves on the perfusion controller. If an M- or X-Series board is used that has four analog outputs such as the PCIe-6323, AnalogOut2 can control a second 8 channel perfusion controller while AnalogOut1 controls the first 8 channel perfusion controller.

For slice experiments and fast perfusion changes during a sweep by a stepper, the inexpensive, slowly operating pinch-valves, with valve changing times of approximately 100 ms, are appropriate. For fast single-line perfusion changes, faster, more expensive valves, such as Lee valves, with 4 ms changing times, are required.

### 2.1 Consideration of *N* for multi-slice experiments

A major consideration when designing a multi-slice experiment with automated perfusion control is: What will your *N* be? This will determine the number of slices in a perfusion chamber, and if more than one, whether the electrical stimulation of these slices will be identical. Many researchers consider that an *N* of 1 is for 1 slice from 1 animal exposed to 1 experimental protocol (including both stimulation and perfusion solutions). So if 1 slice each was obtained from 2 animals, and these two slices were exposed to the *same* stimulation protocol and the *same* perfusion solutions, this would be a ‘strict’ *N* of 2. And if 2 slices were obtained from 1 animal, and these two slices were exposed to the *different* stimulation protocols and *different* perfusion solutions in different chambers, this would also be a ‘strict’ *N* of 2. However, if 2 slices were obtained from 1 animal, and these two slices were exposed to the *same* stimulation protocol and the *same* perfusion solutions, some researchers would consider this to be an *N* of 2, and others would consider it a ‘strict’ *N* of 1. In general we do not favor exposing multiple slices obtained from 1 animal to the *same* stimulation protocol and the *same* perfusion solutions (which would equate to making a single biochemical measurement in, say, triplicate and then averaging to obtain a single *N* value). Similarly, when using expensive drugs or expensive transgenic animals, using more than 1 slice from the same animal and exposing them to the *same* stimulation and perfusion protocol is reasonable because it would increase the probability of a successful experiment, however it would still be a ‘strict’ *N* of 1.

In general, WinLTP was designed to provide, if necessary, completely *different* stimulation protocols and perfusion solutions for one slice in one chamber. Four (and more) instances of WinLTP can be run simultaneously on one computer, and each of these WinLTPs can control completely separate stimulations and perfusions. And taking 4 slices from 1 animal and exposing them to *different* stimulations and perfusion solutions is clearly more difficult to do than taking 4 slices from 1 animal and exposing them to the *same* stimulation and perfusion solution.

## 3 Results

The basic capabilities of WinLTP have been described in detail before (Anderson and Collingridge, 2007). Since the WinLTP version 0.94 of that paper, the following capabilities have been added (as described in www.winltp.com): the ‘RunOnce’ and ‘Run/ElseRun’ events, graph zooming, ‘Maximum Slope’ measurement, series resistance single or double exponential curve fitting, a patch-clamp ‘SealTest Protocol’, analog train and ramp stimulation, converting WinLTP ASCII acquisition sweep files to Axon Binary files, an ‘Experimental Log’, and support for USB M- and X-Series National Instruments boards. In this paper we discuss automated perfusion control, protocol linking, and simultaneously running more than one WinLTP program on one computer.

**Fig. 1:**
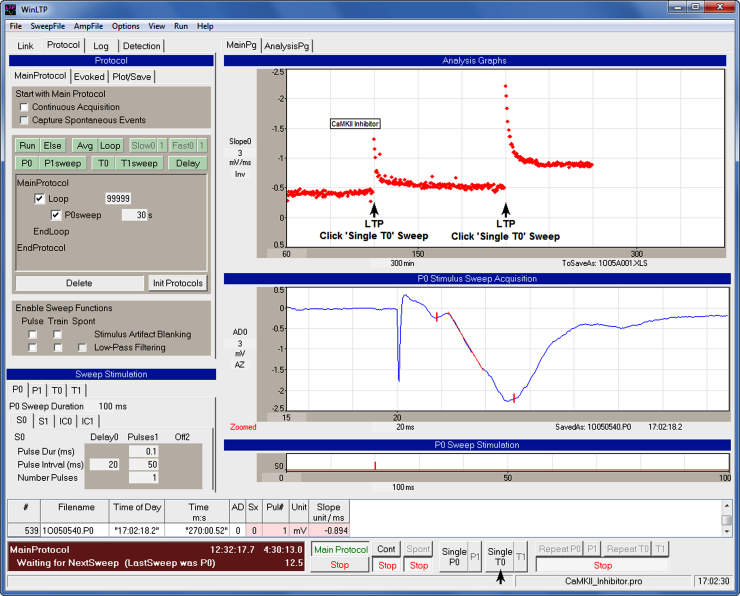
WinLTP layout for a basic LTP experiment showing the ‘Protocol Builder’ (upper left panel), ‘Analysis Graphs’ (in this case only one ‘Slope0’ graph, top right panel), ‘Sweep Acquisition’ (middle right panel), ‘Sweep Stimulation’ fields and graphs (lower left and right panels), and the ‘Spreadsheet’ and ‘Run’ buttons (bottom panels including the ‘Single T0’ Run Button). Detection fields to change analysis of synaptic potentials are hidden. Two LTP induction trains were evoked by twice clicking the ‘Single T0’ Run button (lower black arrow), which evoked two ‘T0sweeps’ (marked by black arrows in the Slope0 graph). The time of CaMKII Inhibitor application is shown by an imposed rectangle in the Slope0 graph.

Fig. 1 shows the layout of the WinLTP program to run a basic LTP experiment showing the ‘Protocol Builder’, ‘Analysis Graphs’, ‘Sweep Acquisition’ graphs, ‘Stimulation’ fields and graphs, ‘Spreadsheet’ and the ‘Run’ buttons. The Protocol Builder shows ‘P0sweeps’ repeating every 30 s, and each P0sweep produced only one S0 extracellular stimulation pulse. The Sweep Acquisition graph shows a field EPSP evoked by S0 stimulation with the slope marked by a red line. Slope calculations are shown by red diamonds in the ‘Slope0’ graph, and also in the ‘Spreadsheet’. Two LTP induction trains were manually evoked by twice clicking the ‘Single T0’ Run button, which produced two 100 S0 pulse T0sweeps (black arrows in the Slope0 graph). A CaM Kinase II Inhibitor was applied before and during the first T0sweep train stimulation (indicated by the rectangle in the Slope0 graph).

### 3.1 From circular to sequential scripting

The basic protocol construct in Fig. 1 is a circular script consisting of a Loop event between the ‘MainProtocol’ and ‘EndProtocol’ lines:

**Figure:**
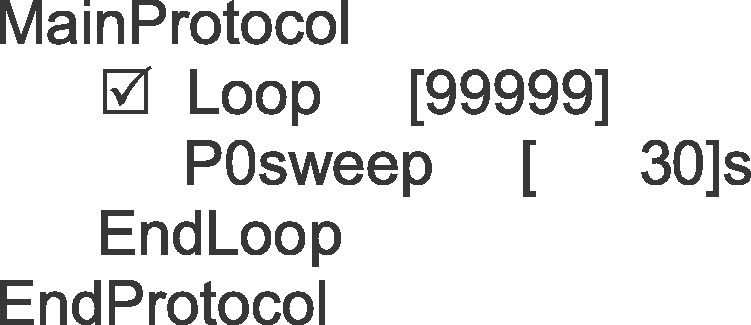

This Loop loops 99,999 times, which means looping continuously for the duration of the experiment, and is therefore called a continuous loop. In circular scripting, a repeating P0sweep (or alternating repetitive P0sweep/P1sweep) event is usually produced by putting a P0sweep (or a P0sweep/P1sweep) between the Loop and ‘EndLoop’ event. This is used to provide baseline stimulation, and constant slow stimulation between induction stimulation and perfusion manipulations. When required, intermittent induction stimulation is delivered by clicking on a ‘Single Sweep’ Run button for LTP induction, or a ‘Repeat Sweep’ Run button for LTD induction. In Fig. 1, the two T0sweep trains were manually evoked by twice clicking on the ‘Single T0’ Run button.

In contrast, sequential scripts follow a linear, not circular, order of events, and this is what experiments really are—a linear sequence of events. While circular scripts are useful for initial investigations when you do not know exactly when you want to stimulate, sequential scripts are better once the timing of the delivery of the stimulation has been designed.

**Fig. 2:**
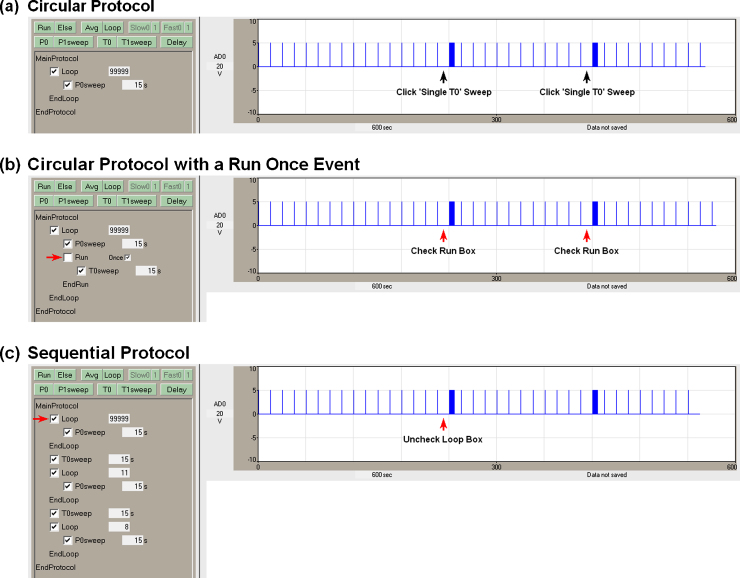
From circular to sequential scripting. This protocol shows a series of non-averaging ‘P0sweeps’ (containing a single S0 pulse, recorded in AD0) separated by two T0sweeps (containing an LTP induction train of 100 S0 pulses). (a) A circular script with a continuous loop of 99,999 loops analogous to Fig. 1. Two single T0sweeps are evoked by twice clicking the ‘Single T0’ sweep Run button (black arrows). (b) A circular script with a ‘RunOnce’ event containing a single T0sweep, where two T0sweeps are evoked by twice checking the ‘Run’ checkbox (red arrows). (c) A sequential script where, after a steady baseline has been determined, the entire stimulation sequence is started by unchecking the Loop checkbox (red arrow). The rest of the protocol runs without the researcher having to be present. (For interpretation of the references to color in this figure legend, the reader is referred to the web version of this article.)

Fig. 2 shows the transformation from circular scripts to sequential scripts with automated perfusion by showing three different protocols that can produce stimulation similar to the LTP experiment shown in Fig. 1. Fig. 2a is a circular script duplicating Fig. 1, with the two T0sweeps manually evoked by clicking on the ‘Single T0’ Run button (black arrows). The researcher has to be present each time to click the ‘Single T0’ sweep button.

Fig. 2b shows a circular script with a RunOnce event containing a single T0sweep. When the ‘Run’ checkbox is checked (red arrows), the events in the RunOnce event are run once. In this case, a single T0sweep will be run once each time the Run checkbox was checked. The RunOnce event also serves as an effective means of intermittently delivering complex induction stimulations (see Fig. 8b,c in Anderson and Collingridge, 2007). The researcher still has to be present each time to check the Run checkbox.

Fig. 2c shows how this protocol can be produced using a sequential script. At the start of the experiment just below the MainProtocol line is the baseline loop section which contains a continuous loop having one P0sweep, which in turn contains one S0 pulse. Because the checkbox of this Loop event is checked, it starts running once the ‘MainProtocol’ button is clicked, and one P0sweep and one S0 pulse is output in each loop. When this Loop checkbox is unchecked (red arrow), this continuous loop is exited, and the next event in the Protocol Builder script is run—a single T0sweep to produce a train of S0 pulses. After this T0sweep, a non-continuous set of 11 loops is run, each containing the P0sweep (to output 11 repetitive S0 pulses). Next the second T0 sweep is run to produce a second S0 pulse train. And finally, after this second T0sweep, another non-continuous set of 8 loops is run to finish up the experiment by outputting 8 more repetitive S0 pulses. With this protocol, the researcher only has to be present to uncheck the continuous ‘Loop’ checkbox (when baseline stability has been achieved), and the rest of the stimulation outputs automatically.

### 3.2 Sequential scripting with automated perfusion control

**Fig. 3:**
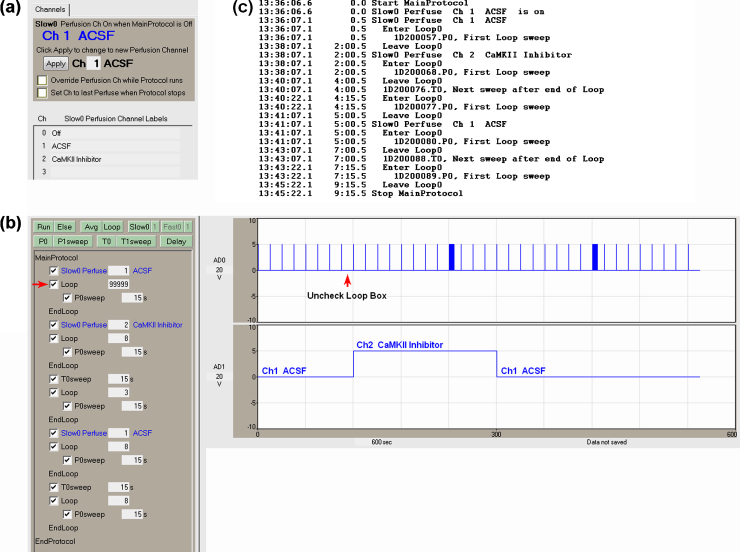
Sequential protocol script with automated perfusion control for a slice experiment. (a) Manual control of the perfusion channels panel (top). Enter the perfusion solution label next to the perfusion channel number (bottom). (b) A sequential script including Perfuse events to change perfusion solutions between sweeps. The entire stimulation sequence is started by unchecking the Loop checkbox (red arrow), and the researcher can leave at this time. (c) The ‘Experimental Log’ output when this protocol is run. (For interpretation of the references to color in this figure legend, the reader is referred to the web version of this article.)

Although sequential scripting is useful when delivering automated electrical stimulation, its major strength becomes apparent is when the automated delivery of electrical stimulation is coupled with the automated change of perfusion solutions. The usage of sequential scripts coupled with automated perfusion control allows almost completely automated experiments to be produced. Fig. 3 shows an example of this for a slice experiment—after achieving baseline stability, switching from Ch1 ACSF to a Ch2 CAMKII Inhibitor solution, delivering an LTP induction train in the CAMKII Inhibitor, switching back to the Ch1 ACSF wash, delivering a second LTP induction train in ACSF, and recording the final responses. This protocol is analogous to the experiment in Fig. 1.

Before starting the experiment, perfusion solution labels are entered next to the perfusion channel number—‘ACSF’ is entered next to Ch1, and ‘CaMKII Inhibitor’ next to Ch2 (Fig. 3a). This causes the labels to appear beside the Perfuse event in the Protocol Builder (Fig. 3b, left) and in the Experimental Log (Fig. 3c).

The perfusion solution can be manually changed when the ‘MainProtocol’ is not running by entering the channel number and clicking on the ‘Apply’ button (Fig. 3a). Furthermore, if you wish to run a MainProtocol with manual control of which perfusion channel is on, or you wish to override the automated perfusion control while the MainProtocol is running, check the ‘Override’ checkbox below the ‘Apply’ button. Alternatively, if you wish for the current perfusion channel to remain on when the MainProtocol finishes, check the ‘Set Ch to Last Perfuse’ checkbox.

At the start of the MainProtocol, the immediate first ‘Slow0 Perfuse’ event makes sure that the experiment starts out perfusing the channel 1 (‘ACSF’) solution. Then the protocol enters a continuous loop with a P0sweep which produces the baseline stimulation. As with the sequential scripting without automated perfusion control (Fig. 2c), when the experimenter has decided that a stable baseline has been reached, this baseline loop is exiting by unchecking the Loop event (red arrow in Fig. 3b). The rest of the stimulation/perfusion change sequence then runs without further manual input.

The first event after the baseline loop is exited is a Slow0 Perfuse event, which changes the perfusion to channel 2 and begins perfusing the CAMKII Inhibitor solution. This is indicated by the bottom trace recording of Bit1 Port1, which shows switching from ‘Ch1’ to ‘Ch2’. Then a second loop containing a P0sweep is run for 8 times, and is followed by the first T0sweep LTP induction stimulation is produced (shown by the first train in the ‘AD0’ recording). Then a third loop containing a P0sweep is run for 3 times, and the Slow Perfuse event changes the perfusion solution back to Ch1 ACSF (shown in the ‘AD1’ recording). Then a fourth loop containing a P0sweep is run for 8 times, followed by the second T0sweep LTP induction stimulation (shown by the second train in the AD0 recording). After this a post induction fifth loop with a P0sweep is run 8 times, and then the MainProtocol ends. The Experimental Log output when this protocol is run (Fig. 3c) clearly shows the Slow0 Perfuse event along with the time, perfusion channel number, either an ‘ACSF’ or ‘CaMKII Inhibitor’ perfusion solution label, and then next sweep following the perfusion change.

### 3.3 Standard and pre-flush automated perfusion of slices

WinLTP can control up to four perfusion lines. Slow0 and Slow1 perfusion change can each control one perfusion line by making only ‘slow’ changes *between* sweeps. However, Fast0 and Fast1 perfusion change can each control one perfusion line by making ‘fast’ changes *during* sweeps as well as *between* sweeps. Normally WinLTP uses Slow0 Perfusion Change for controlling one perfusion line to one slice chamber, and that is what will be used here. However, up to four perfusion line controls (Slow0, Slow1, Fast0 and Fast1) could be simultaneously used for multi-slice experiments.

Mammalian single cell studies in culture typically use HEPES or similar buffer which does not require bubbling with 95% O_2_ and 5% CO_2_. In contrast, mammalian brain slice experiments normally use a bicarbonate buffer bubbled with 95% O_2_ and 5% CO_2_ for correct pH and maintained at 30–37 °C. For slice experiments, when oxygenated/carboxygenated solution has stayed in polyethylene or tygon tubing for a ‘long’ period of time before being perfused on the slice, oxygen can diffuse across the tubing wall. This loss of oxygen also causes changes in solution pH. Delivery of this stale solution during solution changes is one of the main problems of automated perfusion control in slice experiments.

**Fig. 4:**
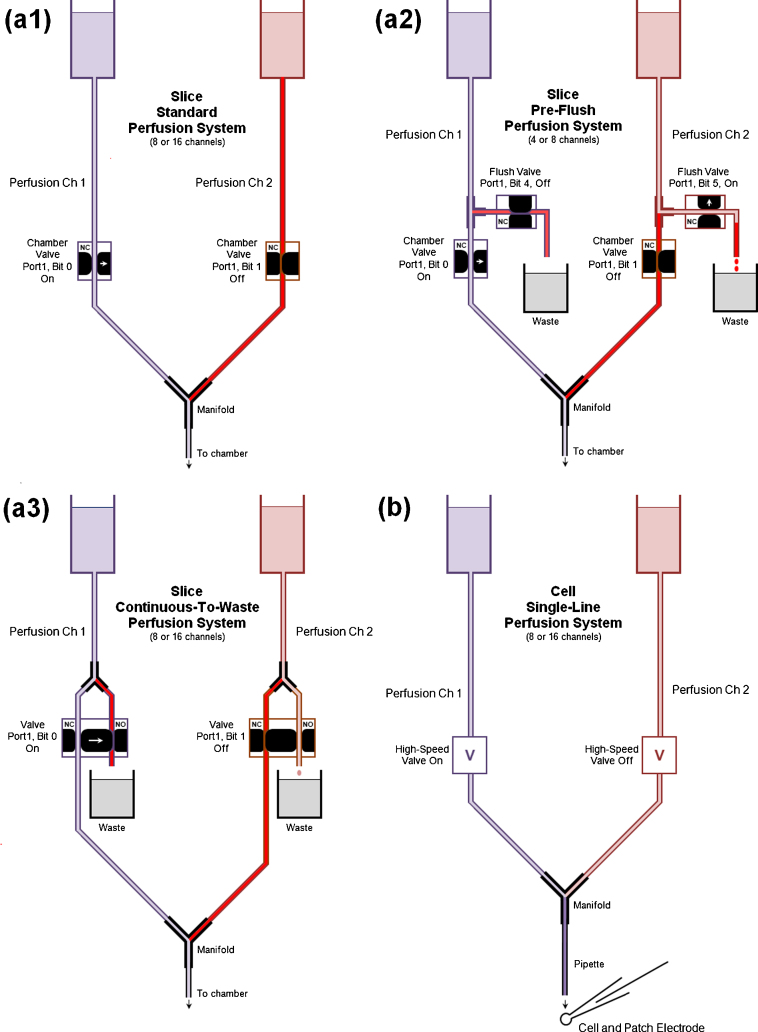
Standard, pre-flush and continuous-flow-to-waste slice perfusion systems (a) and standard single cell single-line perfusion system (b). (a1) The slice standard perfusion system with 1 valve/line. (a2) The slice pre-flush perfusion system with 2 valves/line for a four-line perfusion system. The flush valve has been turned on for a sufficient time to clear the perfusion line from the reservoir to the T-junction, but the valve to the chamber has not yet been turned on. (a3) A continuous-flow-to-waste perfusion system using a Normally Open (‘NO’)–Normally Closed (‘NC’) pinch-valve to flush the tubing between the reservoir and the ‘Y’ junction. The dead volume stale solution in the perfusion lines for these three systems is indicated in red. (b) The standard single-line perfusion system for single cell patch-clamp experiments. The crucial dead volume here is from the manifold to the tip of the perfusion pipette (shown in dark purple). (For interpretation of the references to color in this figure legend, the reader is referred to the web version of this article.)

**Fig. 5:**
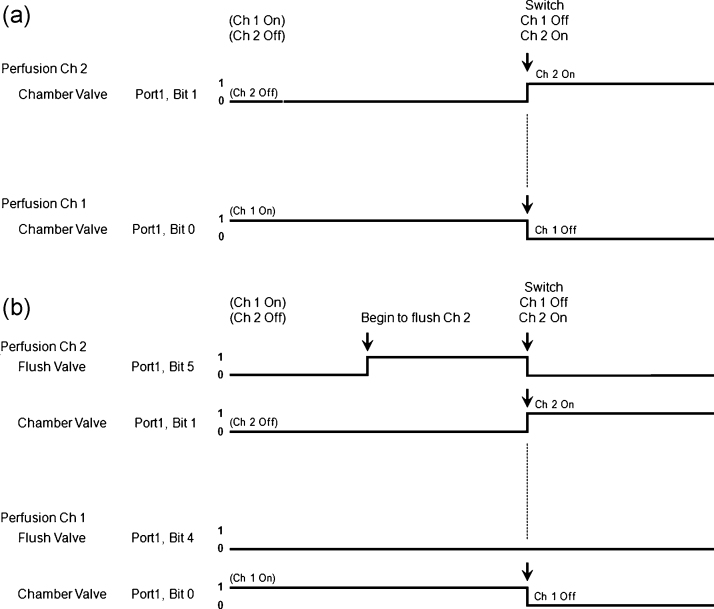
Program output to an automated perfusion controller when switching from Ch1 to Ch2 in a slice experiment when using (a) a standard 8-channel configuration, and (b) a pre-flush 4-channel configuration. Note how the Ch2 Flush Valve (Port1, Bit5) turns on before the switch from Ch1 to Ch2 and then turns off at the switchover.

The standard perfusion system involves 1 valve per perfusion line, and only one valve is open at a time (Fig. 4a1). The perfusion line1/Ch1 valve is open and oxygenated/carboxygenated solution is flowing through the manifold and into the chamber. The solution in perfusion line2/Ch2 is not moving and the stale solution is not being bubbled with O_2_/CO_2_ and the pH is incorrect (indicated by the red solution) because of the bicarbonate buffer. When the solution is switched to perfusion line1 to perfusion line2, the Ch1 valve closes and at the same time the Ch2 valve opens. To do this WinLTP changes Port1 Bit0 from on to off and simultaneously changes Bit1 from off to on (Fig. 5a). All the stale solution in perfusion line2 from the reservoir to the manifold begins flowing into the manifold, and the chamber receives a big bolus of stale solution with incorrect levels of O_2_, CO_2_ and pH.

Obviously, the smaller the volume of solution that ‘sits’ in the perfusion line before being perfused onto the slice, the less effect the stale solution will have on the slice. For slice experiments using standard perfusion, perfusion solution between the oxygenated/carboxygenated reservoir and the manifold (shown in red in Fig. 4a1) is the crucial dead volume solution.

One way to reduce this dead volume in the standard perfusion system is by mounting the syringe reservoir directly on top of the pinch valve, as is possible with some perfusion systems. This makes the perfusion line from the reservoir to the pinch-valve negligible. However, unless you are perfusing room temperature solution, the solution has to be directly warmed by a heater directly surrounding the reservoir syringe. Note also that there is still a short length of tubing between the pinch valve and the manifold that is not oxygenated/carboxygenated.

A second way to reduce the amount of stale solution is for WinLTP to pre-flush the perfusion line between the oxygenated reservoir and just above the valve to the chamber (Fig. 4a2). This is done by inserting a ‘T’ junction just above this valve and having a second waste line go through a flush valve and on to a waste container. The AutoPrime system from Automate Scientific performs this type of pre-flush. In Fig. 4a2 the chamber valve for perfusion line1/Ch1 is open and the flush valve to waste is closed, and oxygenated/carboxygenated solution is flowing into the manifold and on into the chamber. When the solution perfusing the chamber is about to be switched to perfusion line2, the flush valve for perfusion line2 first opens to completely flush the line between the oxygenated/carboxygenated reservoir and the ‘T’ junction, and this solution goes into the waste bottle. Then simultaneously, the chamber valve for perfusion line1/Ch1 closes the flush valve for perfusion line2 closes, and the chamber valve for perfusion line2/Ch2 opens. This causes the small bolus of stale solution in perfusion line2 between the ‘T’ junction and the manifold to go into the manifold and on to the chamber, and this is followed by oxygenated/carboxygenated solution. Thus, in a 4 perfusion line pre-flush system (Figs. 4a2 and 5b), the pre-flush of line2 is started by changing Port1 Bit5 from off to on, then once line2 is flushed, simultaneously changing Bit5 to off to turn off the flush valve of perfusion line2/Ch2, changing Port1 Bit1 to on to turn on the chamber valve of perfusion line2/Ch2, and changing Port1 Bit0 to off to turn off the chamber valve of perfusion line1/Ch1.

Pre-flushing the perfusion line above the chamber valve can substantially reduce the bolus of stale solution entering the chamber, but does not eliminate it. The main drawback to this system is that two valves per perfusion line are required.

A third way to reduce the amount of unoxygenated solution is to use a continuous-flow-to-waste system (Fig. 4a3). With systems that use three-way ‘Normally Closed’–‘Normally Open’ pinch valves, the perfusion line between the reservoir and the manifold is placed in the Normally Closed slot of the pinch valve. A ‘Y’ junction is placed above the pinch valve, and a second perfusion line is connected between this ‘Y’ junction, through the Normally Open slot of the pinch valve and on to a waste container.

When the perfusion channel is off, the pinch valve controlling the solution flow between the ‘Y’ junction and the manifold is Normally Closed and between the ‘Y’ junction and waste is Normally Open, and the tubing between the reservoir and the ‘Y’ junction is filled with oxygenated/carboxygenated solution. When the perfusion channel is turned on, the continuous-flow-to-waste stops, and oxygenated solution now flows from the ‘Y’ junction to the manifold. The dead volume with stale solution here is from the ‘Y’ junction to the manifold, and is indicated in red. The advantage of the continuous-to-waste system is that only one valve per perfusion line is required, the disadvantage is that expensive drug solutions would be wasted.

Estimates of dead volume for 1.32″ (0.8 mm) inner diameter tubing for the pre-flush and continuous-flow-to-waste systems (between the ‘T’ or ‘Y’ junction above the chamber valve to the manifold) are 0.05 ml for 9 cm of tubing in a 4 channel system, and 0.08 ml for 16 cm of tubing in an 8-channel system. The 1/32″ tubing volume (0.05–0.08 ml) is probably negligible for a larger 1 ml chamber, but could be too large for a 0.3 ml chamber.

In addition to these ways of reducing stale solution, other techniques could include: (1) keep the dead volume as small as possible by using as small a diameter tubing and as short a length of tubing as possible between the junction and the manifold, (2) increase the bath volume so that the dead volume is a smaller proportion of the bath volume, (3) increase the flow rate, and (4) use Teflon tubing where the dead volume occurs, and possibly throughout your system. Teflon tubing is less permeable to oxygen diffusing across the tubing wall than polyethylene or tygon tubing.

### 3.4 Automated single-line perfusion patch-camp experiments

In the previous section we have discussed changing perfusion solutions between sweeps during extracellularly recorded multi-slice experiments, and using pre-flush and other techniques to reduce the amount of stale, unoxygenated solution delivered to the slice during solution changes. However, automated perfusion of single cell patch-clamp recording and has a different set of problems. First, single cell patch-clamp experiments use a HEPES or similar type buffer rather than a 95% O_2_/5% CO_2_ bicarbonate buffer, so there is not the concern about stale, unoxygenated altered pH solution in the lines. The dead volume between the reservoir and the manifold is not a crucial concern, and pre-flushing the perfusion lines is not necessary. However, in these experiments, perfusion solutions have to be changed very quickly—sometimes within a millisecond, and so automated perfusion has to be able to be quickly changed both *during* as well as *between* sweeps.

In this and the following sections we discuss two methods of automated perfusion control of single cell patch-clamp experiments. The first method is using a single-line perfusion between the manifold and the end of the pipette near the cell, and solution application lasting 10s of milliseconds to seconds. The second method is to use a stepper to move two or three perfusion tubes, and solution applications can be as short as 1 ms.

The single-line perfusion system is designed to work with the standard, not a pre-flush, perfusion system where 4–16 perfusion lines go into a manifold which then goes into a pipette of approximately 100 μm internal diameter and then onto the cell (Fig. 4b). In these experiments, the biggest problem is the speed of the high-speed valves and dead volume from the manifold to the end of the pipette in the chamber (shown in dark purple in Fig. 4b), because this affects how quickly new perfusion solutions can be changed and reach the cell.

Changing perfusion solutions between sweeps is controlled by the Perfuse event in the Protocol Builder, and fast perfusion change during the sweep is controlled by changing either digital or analog output during the sweep.

For setting up a typical antagonist/agonist single line patch-clamp experiment, a major question is whether or not the antagonist rapidly unbinds. This has a large effect on the number of perfusion solutions (and valves) needed to get a good concentration–response curve. If the antagonist remains bound during the application of an agonist solution, then the antagonist does not need to be added to the agonist solution to get an accurate concentration–response curve. Therefore, you can have separate solutions (and valves) for each agonist solution as well as each antagonist solution. For example, one eight-valve controller is sufficient to do a concentration–response curve for 4 agonist concentrations and 4 antagonist concentrations (one of which could be no antagonist, i.e. ACSF). However, if the antagonist does not remain bound during the application of the agonist-only solution, then the antagonist has to be added to the agonist solution to get an accurate concentration–response curve. Therefore, many more agonist + antagonist solutions (and valves) are required to do a concentration–response curve. For example, to do a concentration–response curve for 4 agonists and only 3 antagonists requires 15 solutions, 15 valves, and one 16 or two 8 channel controllers.

Single-line perfusion can use AnalogOut1 or 4 bits on the high-speed Port0 to control one 8 channel or one 16 channel controller, or it can use two analog outputs (AnalogOut1 + AnalogOut2) to control two 8 channel controllers.

**Fig. 6:**
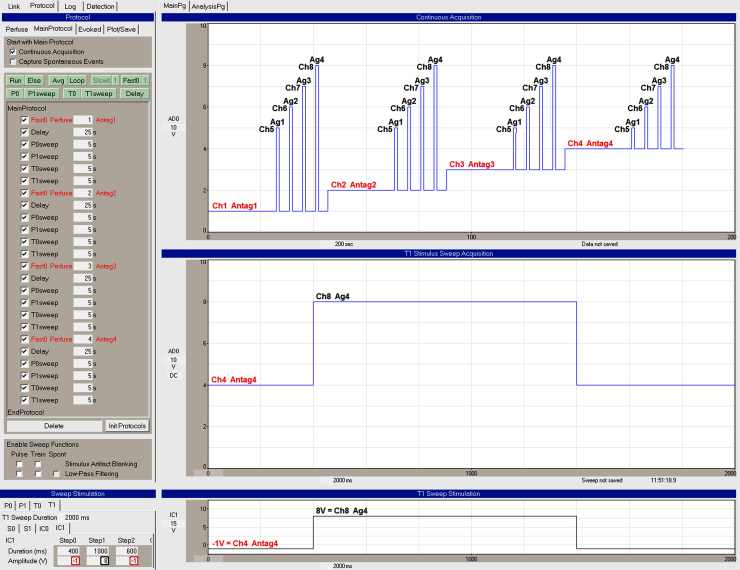
Single cell, single-line fast perfusion changes during and between sweeps—applying 4 different agonists and 4 different antagonists when the antagonist does not rapidly unbind. The ‘Fast0 Perfuse’ events (shown in red in the ‘Protocol Builder’ panel on the left) set the on-going perfusion to Ch1 Antag1, Ch2 Antag2, Ch3 Antag3 and Ch4 Antag4. Four different concentrations of agonist are applied during the 4 different sweeps. During the ‘Step1’ epoch in the T1sweep (bottom panels), a voltage of 8 in the ‘Amplitude (V)’ field (black rectangle) changes the perfusion to Ch 8 or Agonist 4. (For interpretation of the references to color in this figure legend, the reader is referred to the web version of this article.)

An example of single-line fast perfusion changes during and between sweeps is shown in Fig. 6. This is for controlling an 8 channel perfusion controller using AnalogOut1 and to for applying 4 different agonists and 4 different antagonists when the antagonist does not rapidly unbind. The four different agonist concentrations are applied during the four different sweeps. The protocol in the Protocol Builder (left panel) starts with the ‘Fast0 Perfuse’ event causing ‘Antagonist 1’ to be perfused via channel 1. After a delay, ‘Agonist 1’ is applied via channel 5 for 1 s during the P0sweep. Then shortly thereafter ‘Agonist 2’ (channel 6) is applied during a P1sweep, ‘Agonist 3’ (channel 7) is applied during a T0sweep, and ‘Agonist 4’ (channel 8) is applied during a T1sweep. Then the perfusion solution is changed to ‘Antagonist 2’ (channel 2), and application of the 4 agonist solutions is repeated, and this is repeated for ‘Antagonist 3’ perfusion (via channel 3), and finally for ‘Antagonist 4’ perfusion (via channel 4). During the first and third epochs, ‘Step0’ and ‘Step2’, a −1 in the ‘Amplitude (V)’ field (red rectangles) causes the current perfusion solution set by the Fast0 Perfuse event in the Protocol Builder to be maintained in that part of the sweep. During the ‘Step1’ epoch, a voltage from 1 to 8 in the Amplitude (V) field (black rectangle) changes the perfusion to channels 1–8.

Note that if your antagonist does not rapidly unbind, and therefore agonist solutions need not contain antagonist, it is strongly preferable to use AnalogOut1 rather than digital output for fast perfusion changes during a sweep. This is because if you enter a −1 V into the Amplitude (V) field of the epoch, that means that the current fast perfusion channel will continue to be output during sweep epochs containing a −1 V, and therefore each sweep can contain a unique agonist concentration, thereby limiting the number of different sweep stimulations required.

### 3.5 Using protocol linking to extend single-line perfusion

WinLTP currently can output four different sweep stimulations (P0, P1, T0 and T1) from one protocol file. When the antagonist is tightly bound, this means you could deliver 4 agonist concentrations and up to 12 different antagonist concentrations (for up to 16 channels) in one protocol file (when using −1 V and analog output). However, when the antagonist is loosely bound and the agonist solutions must also contain an antagonist, you could deliver only 1 antagonist concentration and 4 agonist concentrations (with that one antagonist concentration) in one protocol file. To test 3 antagonist concentrations and 4 agonist concentrations (or 15 different solutions, 3 antagonist plus 12 agonist + antagonist solutions), you would need to use ‘Protocol Linking’ to link 3 protocol files together. Patch-clamp automated single-line perfusion can realistically involve delivering 15 different perfusion solutions, and 16 channels is a reasonable number of channels going into one manifold. With Protocol Linking, once a protocol has finished, WinLTP can now load and start a second protocol containing 4 different solution changes, and then load and start a third protocol containing 4 further different solution changes, and WinLTP can do this, ad infinitum. The limitation is in the number of perfusion channels available.

**Fig. 7:**
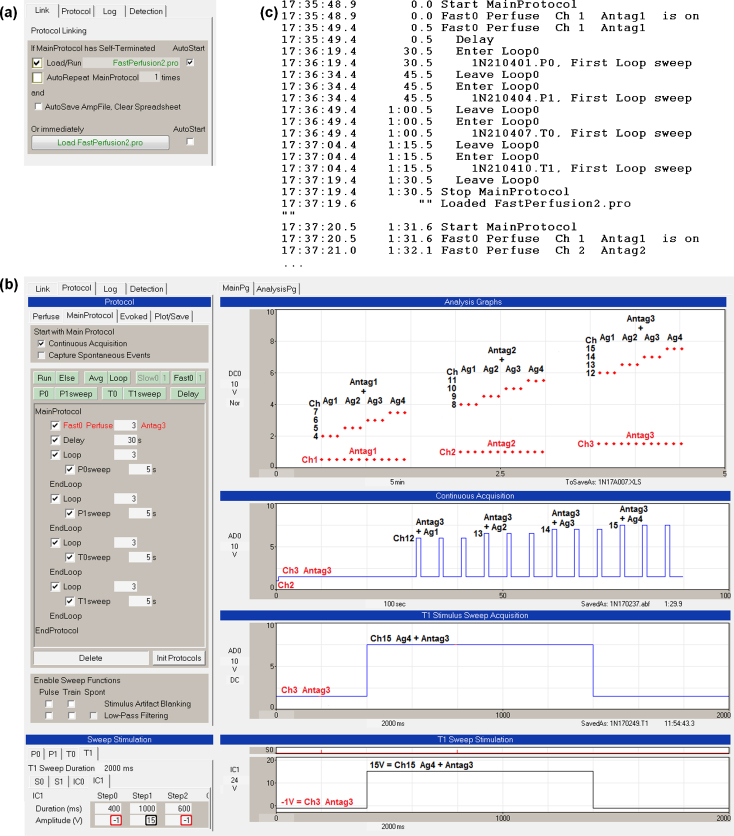
Single cell, single-line perfusion with Protocol Linking. Protocol Linking is used to link 3 different protocol files to continuously perfuse 3 different antagonist concentration solutions, and momentarily apply 4 different agonist concentration solutions also containing the antagonist. A total of 15 different solutions were applied. (a) The ‘Protocol Linking’ tab sheet of FastPerfusion1.pro protocol file which is set to load the next protocol file (FastPerfusion2.pro). Because the ‘AutoStart’ checkbox is checked, FastPerfusion2.pro will automatically start. (b) A screenshot after the third linked protocol file has run. The ‘Analysis Graphs’ records the ‘DC baseline’ voltage of the initial antagonist voltage and the agonist + antagonist voltage for all three linked protocol files. (c) The first part of the ‘Experimental Log’ when the three linked protocols were run. Only the first sweep in a loop is set to be printed to the Log.

Fig. 7 shows a single-line perfusion experiment involving 3 antagonist concentration solutions and 4 agonist concentrations (or 12 agonist + antagonist solutions), where the antagonist is loosely bound to the receptor and has to be added to the agonist solution during agonist application. The ‘Protocol Linking’ tab sheet of the first protocol file (FastPerfusion1.pro) is set to load the next protocol file (FastPerfsuion2.pro) when the FastPerfusion1.pro MainProtocol has self-terminated. Also, because the AutoStart checkbox is checked, the second protocol file automatically starts (Fig. 7a). So once the FastProtocol1.pro protocol runs and self-terminates, FastProtocol2.pro loads, auto starts, runs and self-terminates, and then FastProtocol3.pro protocol loads, auto starts, runs and self-terminates, and the experiment is then ended (Fig. 7b).

Fig. 7b shows the results after the third linked protocol file has finished. The first protocol file (FastProtocol1.pro) delivered ‘Antagonist 1’, then ‘Antagonist 1 + Agonist 1’, then ‘Antagonist 1 + Agonist 2’, then ‘Antagonist 1 + Agonist 3’, then ‘Antagonist 1 + Agonist 4’. The second protocol file (FastProtocol2.pro) delivered ‘Antagonist 2’, then ‘Antagonist 2 + Agonists 1 to 4’, and then the third protocol file (FastProtocol3.pro) delivered ‘Antagonist 3’, then ‘Antagonist 3 + Agonists 1 to 4’.

The Protocol Builder (large panel on left) shows that when the third protocol file auto-starts, the Perfuse event causes ‘Antagonist 3’ solution to be continuously perfused (via channel 3). Then four different sweeps (P0, P1, T0 then T1) are output 3 times. Each sweep contains a 1000 ms output of ‘Antagonist 3 + Agonist 1’ (P0sweep, channel 12), ‘Antagonist 3 + Agonist 2’ (P1sweep, channel 13), ‘Antagonist 3 + Agonist 3’ (T0sweep, channel 14) and ‘Antagonist 3 + Agonist 4’ (T1sweep, channel 15, shown in the bottom two panels). For the T1sweep, setting the Step1 Amplitude (V) to 15 V turns on channel 15 perfusion (bottom left panel, black rectangle). The top right Analysis Graphs panel shows DC output voltages for turning on the channels 1–3 antagonist solutions and channels 4–15 antagonist + agonist solutions for the first, linked second, and linked third protocol files. The ‘Continuous Acquisition’ panel shows the output voltage for the third protocol file only. In this way, 1 antagonist solution and 4 agonist concentrations plus the antagonist for each protocol file, or 3 antagonist solutions and 12 agonist + antagonist solutions, or a total of 15 solutions were tested in the experiment.

The Experimental Log for these linked protocols (Fig. 7c) shows starting the first protocol (FastPerfusion1.pro) with ‘Antag1’—up to the loading and starting of the second protocol (FastPerfusion2.pro) and the switching to ‘Antag2’.

### 3.6 Automated dual- and triple-line/stepper perfusion patch-clamp experiments

Slice automated perfusion control is used to produce solution changes in the minutes timeframe. Single-line automated perfusion control for patch-clamp experiments is used to produce solution applications in the tens of milliseconds to seconds timeframe. In this section we implement perfusion applications as short as a millisecond in duration by using a stepper along with two or three valve controllers. In contrast to single-line perfusion, which requires using fast, expensive valves, the dual- and triple-Line/stepper perfusion system needs to use only the slower, inexpensive pinch valves used with slice perfusion.

**Fig. 8:**
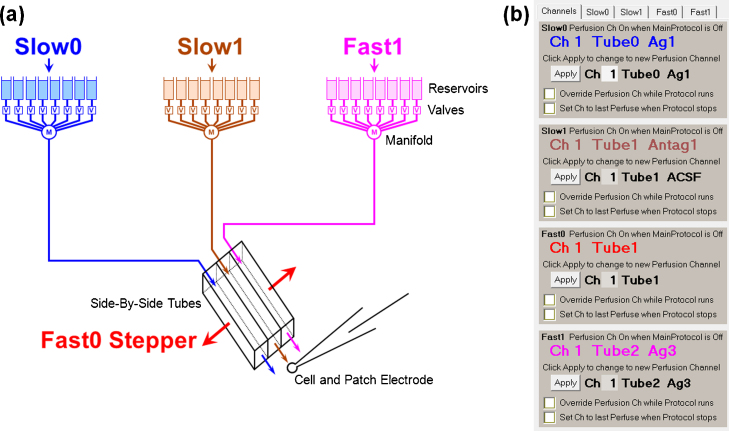
WinLTP control of triple-line perfusion using a Stepper. (a) Slow0, Slow1 and Fast1 Perfusion control three valve controllers, and the Fast0 Perfusion controls the stepper which controls whether the solution from Tube0 (blue), Tube1 (brown) or Tube2 (fuchsia) bathes the cell. The Fast0 Stepper therefore controls whether antagonist (Slow1) or agonist (Slow0 or Fast1) is applied. (b) The panel showing manual control of the color coded Slow0, Slow1, Fast0, and Fast1 perfusion channels. (For interpretation of the references to color in this figure legend, the reader is referred to the web version of this article.)

WinLTP's control of a triple-line/stepper perfusion system is shown in Fig. 8. Slow0 and Fast1 perfusion each controls one perfusion controller, each of which in turn usually controls 4–16 valves. Slow0 and Fast1 usually output agonist or agonist + antagonist solutions from Tube0 and Tube2, respectively. The Slow1 perfusion controls another perfusion controller which in turn controls 4–16 valves. Slow1 usually outputs antagonist only solutions from Tube1. The Slow0, Slow1 and Fast1 perfusion changes solutions *between* sweeps. The Fast0 perfusion controls the stepper which quickly moves the antagonist only Tube1 to either agonist (+antagonist) Tube0 or Tube2 *during* the sweep. If the stepper is a fast piezo device, solution applications from Tube0 or Tube2 can be as quick as one millisecond in duration. The only difference between dual- and triple-line/stepper perfusion is that dual-line perfusion does not use Fast1 perfusion or Tube2.

As with single-line perfusion, when designing your experiment you have to determine whether the antagonist is strongly or weakly bound to the receptor. If the antagonist is strongly bound and is not displaced during agonist application, then as minimal set of agonist and antagonist solutions is required to produce a good concentration–response curve. If the antagonist is weakly bound and is displaced during agonist application, then a much larger set of agonist + antagonist solutions is required to get a good concentration–response curve. For triple-line/stepper perfusion, WinLTP can easily control 16 valves and solutions by Slow0, Fast1 and Slow1 each, so WinLTP can easily control 16 concentrations of antagonist solutions (Slow1) and 32 concentrations of agonist (+antagonist) solutions (Slow0 and Fast1). The problem is more cost of the valves and controllers, and the difficulty in setting up that many perfusion lines rather than with limitations in WinLTP.

**Fig. 9:**
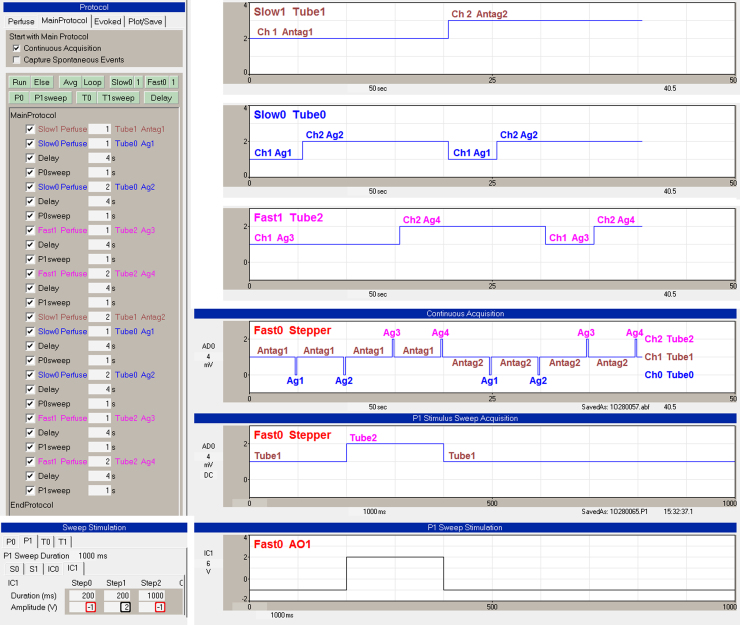
Triple-line automated perfusion with stepper control. The ‘Slow0 Perfuse’, ‘Slow1 Perfuse’ and ‘Fast1 Perfuse’ events in the ‘Protocol Builder’ (upper left) show when the Slow0/Tube0, Slow1/Tube1 and Fast1/Tube2 solutions are changed. The upper right panel shows the Slow1/Tube1 switch from Ch1 Antag1 to Ch2 Antag2. The next panel below shows the Slow0/Tube0 switch between Ch1 Ag1 and Ch2 Ag2. The next panel below that shows the Fast1/Tube2 switch between Ch1 Ag3 and Ch2 Ag4. The Fast0 Stepper ‘Continuous Acquisition’ and the Fast0 Stepper ‘P0 Stimulus Sweep Acquisition’ panels show recordings of the AnalogOut1 voltage momentarily changing from Ch1 Tube1 to Ch0 Tube0 or to Ch2 Tube2. The ‘Sweep Stimulation’ bottom panels show the change of AnalogOut1 (‘IC1’) voltage output during a P1sweep from 1 V or Ch1 Tube1 to 2 V or Ch2 Tube2. The −1 V (red rectangles) means to take the current Fast0 output *between* sweeps, e.g. Ch1 Tube1. (For interpretation of the references to color in this figure legend, the reader is referred to the web version of this article.)

Fig. 9 shows a protocol involving three perfusion lines and side-by-side tubes, Tube0, Tube1 and Tube2, controlled by Slow0, Slow1 and Fast1 perfusion, respectively. Fast0 controls a stepper to switch from Tube1 to Tube0, or Tube1 to Tube2. The ‘Slow0 Perfuse’, ‘Slow1 Perfuse’ and ‘Fast1 Perfuse’ events in the Protocol Builder (upper left) show when the Slow0/Tube0, Slow1/Tube1 and Fast1/Tube2 solutions are changed.

The MainProtocol starts out with the Slow1 Perfuse event making sure the Slow1/Tube1 controller is switched to ‘Antag1’. The Slow0 Perfuse event makes sure the Slow0/Tube0 controller is switched to ‘Ag1’, and then switches to ‘Ag2’. The Fast0/Stepper momentarily switches from ‘Ch1 Tube1’ to ‘Ch0 Tube0’ twice to momentarily switch from ‘Antag1’ to ‘Ag1’ and then from ‘Antag1’ to ‘Ag2’ as shown in the Fast0 Stepper Continuous Acquisition panel. The Fast1 Perfuse event makes sure the Fast1/Tube2 controller is switched to ‘Ag3’, and then switches to ‘Ag4’. Then the Fast0/Stepper momentarily switches from ‘Ch1 Tube1’ to ‘Ch2 Tube2’ twice to momentarily switch from ‘Antag1’ to ‘Ag3’ and then from ‘Antag1’ to ‘Ag4’. Then the Slow1 Perfuse event switches to ‘Antag2’ and then ‘Ag1’, ‘Ag2’, ‘Ag3’ and ‘Ag4’ were similarly delivered in ‘Antag2’ as shown in the right side of the Continuous Acquisition panel.

## 4 Discussion

The addition of automated perfusion change to the automated stimulation and acquisition provided in the Protocol Builder in WinLTP goes a substantial way toward enabling fully automated experiments. The automated perfusion of slice experiments substantially decreases the difficulty of running a single slice/single chamber experiment. Furthermore, because many WinLTP programs can be run simultaneously on one computer, and because the cost of National Instruments boards and WinLTP Advanced Mode software is low, it becomes feasible to run many independent single slice/single chamber experiments simultaneously. Automated perfusion that includes perfusion changes *between* sweeps also aids in the ease of running single cell patch clamp experiments.

Because HEPES or similar buffer is used for single cell patch-clamp experiments, there are no additional problems with delivering solutions using the single-line, or the dual- or triple-line/stepper automated perfusion. However, mammalian brain slices are usually bathed in warmed bicarbonate buffered solutions that must be bubbled with 95% O_2_/5% CO_2_ to maintain the correct oxygen and pH levels. This means that stale solution in the perfusion line between the reservoir and the manifold will be delivered when the perfusion is changed to a new solution. Some, and maybe most, of this stale solution can be removed by pre-flushing the line between the reservoir and a ‘T’ junction above the valve to the chamber using either a two valve pre-flush system such as the Automate AutoPrime system, or a continuous-flow-to-waste one valve system. Or the reservoir can be placed directly above the valve. However, the approximate 9 cm of tubing of approximately 0.05 ml between the ‘T’ junction and the manifold will still contain stale solution. The effect of this stale solution can be further minimized but not eliminated by using smaller diameter tubing, using as short a length of tubing between the junction and manifold, using a larger volume chamber, increasing the flow rate, and using Teflon tubing.

There are several commercial multi-slice systems available (Dunlop et al., 2008). The SynchroSlice from Lohmann Research (www.lohres.com) performs conventional extracellular microelectrode recording for a 4 slice/4 chamber, or 8 slice/8 chamber system. Furthermore, its SynchroBrain software is the only multi-slice software, other than WinLTP, that integrates direct control automated perfusion into the program stimulation. The Scientifica SliceMaster (www.scientifica.uk.com) is a four slice/4 chamber, or eight slice/8 chamber system that uses conventional extracellular microelectrode recording. It can be used with either Cambridge Electronic Design Spike2 based software (Stopps et al., 2004) or Notocord based software (Kroker et al., 2011). The Notocord software does not directly control automated perfusion, but instead uses a Scientifica program that runs in parallel and that directly controls when and what automated perfusion changes take place. The Spike2 software does not control automated perfusion. MultiChannel Systems has developed a system, USB-MEA32-STIM4, that can record from brain slices using a planar 32 channel multi-electrode array system, and their LTP-Director software performs LTP experimental protocols and analyses. Several of these systems could be employed together to produce a similarly priced multi-slice system. The LTP Director software does not control automated perfusion.

For the single cell patch-clamp experiments, most electrophysiological software enables changes to perfusion systems during the sweep by changing analog or digital output during the sweep. In addition to WinLTP, there are, to our knowledge, two patch clamp electrophysiological programs that enable changes between as well as during sweeps—Heka's PatchMaster and Molecular Device's pClamp. PatchMaster has a Protocol Editor that works similarly to WinLTP's Protocol Builder. Perfusion changes are made by inserting ‘Set DAC’ and ‘Set Digital Word’ events into the Protocol Editor script, which directly control the perfusion controllers between sweeps by changing an analog voltage or a 16-bit digital word. pClamp uses a Sequence Key List to set the correct analog or digital output to change perfusion solutions between sweeps.

In contrast, automated perfusion is fully integrated into the WinLTP program. Perfusion changes between sweeps are made in WinLTP by inserting a Perfuse event into the Protocol Builder script and setting the correct perfusion *channel number*. WinLTP also prints the associated *solution label* in the Perfuse event, and automatically converts the channel number to the correct analog voltage or digital output. This allows easy control of perfusion controllers, especially when using 4-bit binary digital output, or two analog outputs. This perfusion change information is also printed to the Experimental Log. These capabilities make it more obvious when and to what the solution is being changed to. Furthermore, the perfusion changes are clearly linked in time with the neural stimulation, which is not the case if the stimulation/acquisition program is running parallel with a separate perfusion program. And finally, WinLTP is the only electrophysiology program that supports pre-flushing for slice experiments.

There is one additional perfusion system which is useful when there is a minimal amount of solution—recirculating the waste from the slice chamber back to the original reservoir. WinLTP can implement this by using one Perfuse event (e.g. Slow0) to control which solution to perfuse, and a second Perfuse event (e.g. Slow1) to control whether the chamber waste solution is recirculated back to the original reservoir, or is sent to the waste container.

Although the addition of automated perfusion in the Protocol Builder goes a long way toward fully automated experiments, there are several limitations in WinLTP that bar the complete implementation of this. First, for extracellularly recorded slice experiments, there is currently no increment/decrement capability for changing extracellular stimulus amplitude when using a voltage-controlled stimulus isolator, and so there is no way of running an automated input–output curve. Second, there is no automated way of determining when a stable baseline is achieved so that the rest of the experiment can be run. Instead, the researcher must unclick the initial continuous loop to run the rest of the experiment. Third, choosing the correct stimulus amplitude still has to be done manually. However, running the input–output curve, determining the correct stimulus amplitude, and determining when a stable baseline is achieved, are normally performed at the beginning of the experiment, at least for LTP and similar synaptic plasticity experiments. This allows most of this type of experiment to be automated. For patch-clamping experiments, the absence of increment/decrement intracellular stimulation capability is a similar drawback. Addition of increment/decrement stimulation and automated detection of stable baselines are planned in future versions of WinLTP, although there are no plans to automate determination of the correct stimulus amplitude.
